# An objective spinal motion imaging assessment (OSMIA): reliability, accuracy and exposure data

**DOI:** 10.1186/1471-2474-7-1

**Published:** 2006-01-04

**Authors:** Alan C Breen, Jennifer M Muggleton, Fiona E Mellor

**Affiliations:** 1 Institute for Musculoskeletal Research and Clinical Implementation, Anglo-European College of Chiropractic, 13-15 Parkwood Road, Bournemouth, BH5 2DF, UK; 2 Institute of Sound and Vibration Research, University of Southampton, Southampton, SO17 1BJ, UK

## Abstract

**Background:**

Minimally-invasive measurement of continuous inter-vertebral motion in clinical settings is difficult to achieve. This paper describes the reliability, validity and radiation exposure levels in a new Objective Spinal Motion Imaging Assessment system (OSMIA) based on low-dose fluoroscopy and image processing.

**Methods:**

Fluoroscopic sequences in coronal and sagittal planes were obtained from 2 calibration models using dry lumbar vertebrae, plus the lumbar spines of 30 asymptomatic volunteers. Calibration model 1 (mobile) was screened upright, in 7 inter-vertebral positions. The volunteers and calibration model 2 (fixed) were screened on a motorised table comprising 2 horizontal sections, one of which moved through 80 degrees. Model 2 was screened during motion 5 times and the L2-S1 levels of the volunteers twice. Images were digitised at 5fps.

Inter-vertebral motion from model 1 was compared to its pre-settings to investigate accuracy. For volunteers and model 2, the first digitised image in each sequence was marked with templates. Vertebrae were tracked throughout the motion using automated frame-to-frame registration. For each frame, vertebral angles were subtracted giving inter-vertebral motion graphs. Volunteer data were acquired twice on the same day and analysed by two blinded observers. The root-mean-square (RMS) differences between paired data were used as the measure of reliability.

**Results:**

RMS difference between reference and computed inter-vertebral angles in model 1 was 0.32 degrees for side-bending and 0.52 degrees for flexion-extension. For model 2, X-ray positioning contributed more to the variance of range measurement than did automated registration. For volunteer image sequences, RMS inter-observer variation in intervertebral motion range in the coronal plane was 1.86 degreesand intra-subject biological variation was between 2.75 degrees and 2.91 degrees. RMS inter-observer variation in the sagittal plane was 1.94 degrees. Radiation dosages in each view were below the levels recommended for a plain film.

**Conclusion:**

OSMIA can measure inter-vertebral angular motion patterns in routine clinical settings if modern image intensifier systems are used. It requires skilful radiography to achieve optimal positioning and dose limitation. Reliability in individual subjects can be judged from the variance of their averaged inter-vertebral angles and by observing automated image registration.

## Background

The measurement of inter-vertebral motion in clinical settings has been a challenge to the field of biomechanics for many years. Early work that sought to use X-rays for kinematic measurement [1-3] first contented itself with qualitative assessment, but gradually moved toward seeking objective measurement. This was largely driven by the clinical imperative to add objectivity to the understanding of what was termed 'instability' [4,5], and which still remains unclear despite a large volume of practical and theoretical research [6-16].

The increasing use of low back stabilisation surgery in the United States over the past 20 years [17] and the rate of re-operations [18] has also made it important to understand and measure lumbar spine motion in patients. Many of the latter procedures are carried out suspecting subtle pseudarthrosis, which is poorly detected by plain radiography [19]. More sophisticated imaging methods, such as Computed Tomography (CT), are able to demonstrate the presence of bony trabeculae across the fusion site but suffer degradation of image quality if metal implants are used. Other imaging methods include Single Positron Emission Computed Tomography (SPECT), however the sensitivity and specificity of SPECT alone is insufficient to diagnose pseudarthrosis [20]. Kinematic evaluation of actual fusion techniques is generally restricted to cadaveric studies [21]. Clinical assessments, however, are needed. The investigation of mechanical derangements at segments adjacent to stabilised ones also requires an *in vivo *technique [22-25], and the rationale for new flexible stabilisation systems depends on understanding how this manifests in patients [26].

Inter-vertebral motion analysis *in vivo *is needed to inform clinician and patient choices about continued conservative or initial surgical treatment for intractable chronic back pain. Subgroups that may do better with one or the other might be revealed if motion patterns were quantifiable and could be evaluated against clinical outcomes. So far, the evidence for lumbar spine stabilisation surgery is conflicting [27] and recent large trials have deepened this uncertainty [28,29], making greater the need for improving our understanding of how stabilisation works and how it affects adjacent levels.

The means to measure inter-vertebral kinematics *in vivo *have improved, but there are still limitations. Zhang and Xiong [30] experimented with applying kinematic models of inter-vertebral motion to external markers for an indirect means of measurement of centres of rotation, but did not establish its reliability. Johnsson et al. [31] used a roentgen stereophotogrammetric method and Harada et al. [32] used cineradiography, but neither has become a clinical tool owing to invasiveness or high radiation dosage. Cheung et al. [33] researched the reliability of digital imaging for measuring Cobb angles in scoliotics but did not assess motion. Zheng et al. [34] used edge extraction from fluoroscopic images to visualise lumbar vertebral outlines for use in animations, and Teyhen et al. [35] demonstrated good intra-observer and intra-subject biological variation in using such a technique for point placement with a screen cursor to calculate lumbar inter-vertebral motion ranges between 2 positions. Murata et al. [36] compared magnetic resonance images and plain radiographs in an attempt to shed light on lumbar segmental instability, and dynamic MRI images from open coil systems were used by McGregor et al. [37] to investigate posterior-anterior mobilisation therapy and by Wardlaw (personal communication) to determine ranges of motion in surgical patients.

### Digitising from fluoroscopy

In the late 1980s, our group found that digitised videofluoroscopic images of the lumbar spine could be used in sequence to measure inter-vertebral motion patterns by assigning co-ordinates to landmarks on each vertebral image with a screen cursor [38-40]. This was replicated by Cholewicki et al. [41] and by Lee et al. [42]. The technique was subsequently used to study the synchronicity of motion between vertebrae during weightbearing in sidebending [43], in flexion-extension [44] and in clinical studies [45]. However, the manual marking of a sufficient number of vertebral images to objectively measure patterns made the technique too laborious for routine use. Automated registration of vertebrae was attempted but, with the image quality available, this was only achievable in a calibration model [46]. Finally, patient motion during active bending was too unstandardised to allow interpretation of the inter-vertebral whole motion sequences that were obtained. There was therefore a need to develop a technique that employs controlled trunk motion, together with automated frame-to-frame registration of vertebral position to allow meaningful and routine clinical use of this technology. The present paper reports the results of work supported by the Department of Health's New and Emerging Applications of Technology Programme (NEAT) that has resolved these difficulties and made available an Objective Spinal Motion Imaging Assessment (OSMIA) for routine use in fluoroscopy rooms.

## Methods

### Patient data acquisition

**Figure 1:**
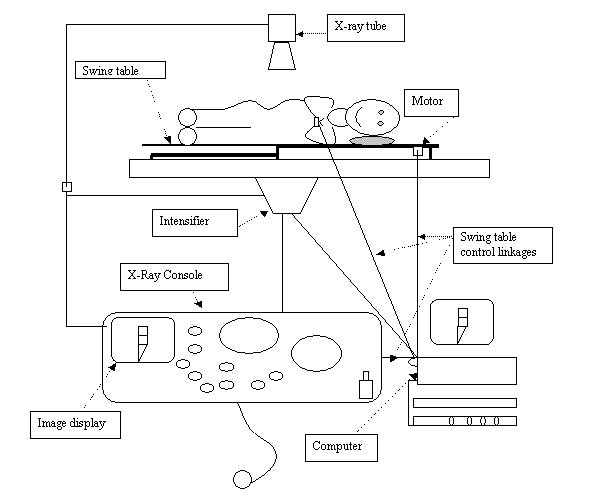
The OSMIA image acquisition system.

**Figure 2:**
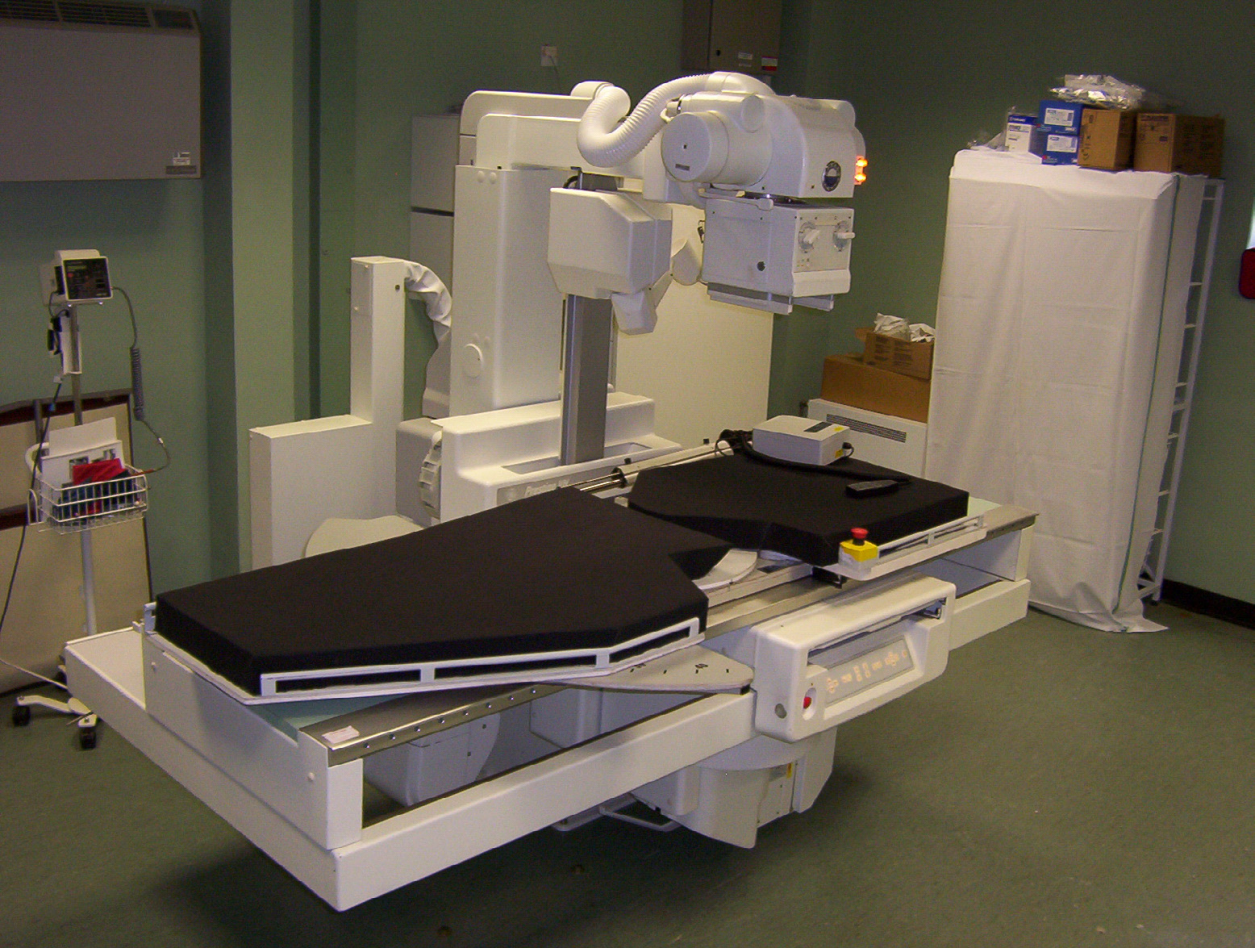
Swing table and fluoroscope configuration. (Atlas Clinical Ltd)

The OSMIA acquisition system (Figure 1) consisted of a portable passive motion table clamped to an X-ray fluoroscope table (GE Systems Prestige Fluoroscope Unit). Analogue images from the fluoroscope were accessed at 5 frames per second by a PC fitted with a framegrabber and time-code generator. The passive motion table (Atlas Clinical Ltd. Figure 2) had a lower section that could execute a smooth arc from the neutral position to 40° left, then to 40° right and back to neutral in one motion. This was driven by a motor controlled from behind the X-ray console. The sequence took 20 seconds to execute, plus a maximum of 4 seconds for positioning.

After giving written informed consent, 30 male volunteer subjects, aged 18–40 and with no back problems in the previous year, were screened lying relaxed on the passive motion table in the coronal, then in the sagittal plane at a focus-to-intensifier distance of 1 m. They were then released for 1/2 hour to move at leisure around the X-ray department waiting area, after which they were screened again. Screenings employed gonadal protection to reduce patient dose and lead shielding to reduce intensifier flare. Exposure data, height and weight were recorded. (All patient data were acquired under protocols approved by the Salisbury Local Research Ethics Committee and Salisbury District Hospital Management Approval)

To optimise relaxation, subjects were allowed to experience the motion before the actual screening. Prior to screening, the central X-ray beam, the L4-5 disc space and the centre of the arc of the swing table were aligned and the patient centred during a brief exposure. After a countdown, the radiographer began the exposure and a second technician began the image acquisition and table motion sequence. The framegrabber acquired approximately 120 tiff files (representing 24s in real time) into computer RAM during the sequence. These were subsequently downloaded onto the hard drive for later analysis.

### Image analysis

**Figure 3:**
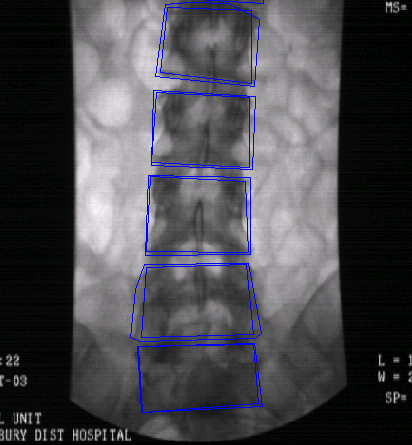
First digital image in a side bending sequence, cursor-marked with external (tracking) and internal (visual) templates.

The image sequences were analysed by 2 observers blind to each other's results until all data were analysed. An automated analysis procedure, executed in MATLAB (The Mathworks Ltd), was used to locate the vertebrae in each successive frame of the motion sequence once they had been manually identified in the first frame. This required two templates to be drawn around each vertebra in the first frame: one simply to define reference points (typically vertebral corners) and one intended to enclose each vertebral body in its entirety (Figure 3). The automated analysis calculated the absolute position and orientation of each vertebra in each frame, but only the orientations (i.e. vertebral angles relative to the computer's x-axis) were used in subsequent analysis. The template marking process was repeated 5 times so that the results could be averaged.

### Intensifier distortion correction

A 300 mm square aluminium grid with 1-cm squares was placed against the image intensifier and X-rayed. The displacements of the corners of the squares on the image due to intensifier distortion were used to write corrective transformations that were applied to the subject images prior to analysis.

### Dose measurement

For the measurement of effective dose, an X-ray phantom fitted with dosimeters was subjected to 30 seconds of screening in the coronal and sagittal planes at 73 KV and 2 mA.

### Calibration studies 1

**Figure 4:**
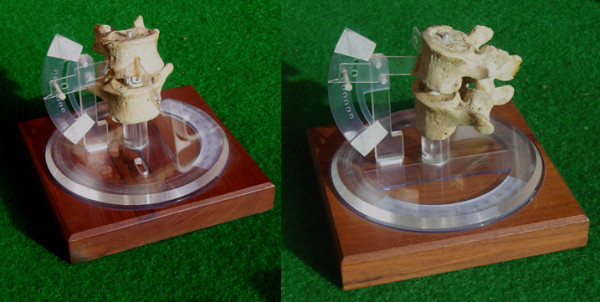
Calibration model 1. with inter-body universal joint and calibrated protractors for intervertebral angle and whole model rotational settings

Calibration model 1 (Figure 4) consisted of 2 human lumbar vertebrae, (L3 and L4) fitted with protractors and joined together with an inter-body universal joint. These provided 7 settings at 5° intervals from -10° to + 20° and could be detached so that coronal and sagittal plane rotations of the superior vertebrae on the inferior one could be measured interchangeably.

With the X-ray table in the upright position, the model was clamped to the table footplate, 5 cm from the intensifier and surrounded on all sides with packets of sausages. This soft tissue was used to degrade the images in a way similar to a living subject, where X-ray scatter and bowel gas can challenge the process of marking bony landmarks with a screen cursor. Fluoroscopic exposures were digitised in optimal and degraded conditions in the 7 model positions in each plane and removed for analysis. The optimal condition was represented by orthogonal alignment of the model to the X-ray beam and the degraded condition by the model being axially rotated 10° out of plane and the X-ray beam inclined 10° inferiorly.

### Calibration studies 2

**Figure 5:**
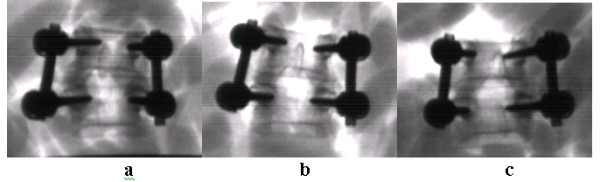
Digitised fluoroscopic images of calibration model 2 right rotated (a), neutral (b) and left rotated (c).

In order to determine the contribution of vertebral template marking error relative to that contributed by radiographic distortion due to scoliosis or mal-positioning, 2 dry human lumbar vertebrae were fixed rigidly together in the neutral position with pedicle screws and metal rods (Calibration model 2 – see Figure 5). These were also surrounded with packs of sausages and re-placed on the motion table as in a patient acquisition procedure, with no attempt to keep alignment orthogonal by any other means than manual placement. The table was then moved through 80° while screening. The table motion was smooth and even and the weight of the model and surrounding soft tissues were enough to stabilise it. This acquisition procedure was done 5 times to simulate the range of axial rotation in positioning that might happen in real life. Each sequence was analysed 5 times and the variance of the ranges of inter-vertebral motion compared to the variance of ranges between runs. (Any range of inter-vertebral motion was error, since the true range of motion was zero degrees). This was done with high (75 KV) and low (65 KV) exposures and with image bit depth set at 8 and 10-bit. Low KV techniques provide more contrast and may therefore provide more reliable analysis. Ten-bit images give twice the contrast (dynamic range) as 8-bit ones and might be expected to do the same.

#### Human subject data collection and data analysis

Image acquisition from all subjects did not require any restraint or stabilisation on the passive motion table as all subjects were able to tolerate the full 80° arcs relaxed and in comfort.

**Figure 6:**
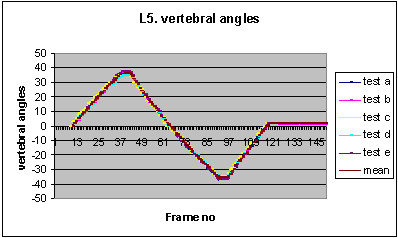
Example of a graph that met the criteria for entry into pool of intervertebral motion graphs; 5 consecutive trackings (relative to the computer's X-axis) of a series of vertebral angles that coincide visually. (acquisition at 5 f/s for 8-bit images)

From the downloaded image files, vertebral angles (relative to the computer's X-axis) from each of the 5 individual vertebral markings of the human subject sequences were all plotted throughout the motion. An example of this is shown (Figure 6). All the graphs start off from zero and because the template assigned to the first image is used throughout the sequence, the results are independent of which vertebral landmarks are chosen to define this template in the first instance.

**Figure 7:**
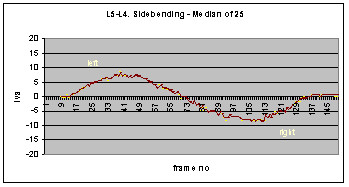
Example from intervertebral motion graph pool. Solid line is median of 25 differences between adjacent vertebral angle series' that met the criteria for inclusion. Scatter plot represents all data points. (acquisition at 5 f/s for 8-bit images).

Only graphs in which all vertebral angles in the 5 runs coincided visually were regarded as reliable and therefore entered into the data pool. Two observers independently inspected all graphs for inclusion. Only those that met this criterion and were adjacent to vertebrae whose graphs did too, were analysed. The analysis consisted of subtracting the vertebral angle sequences of adjacent segments from each other in all combinations (i.e. L1a-L2a, L1a-L2b, L1b-L2b etc) to give inter-vertebral angles throughout the motion. This gave 25 individual inter-vertebral angles for each of 120 images in each motion sequence. These were represented graphically with the median as a solid line and each of the individual 25 points as a scatter plot (Figure 7), showing the full range and variation of each vertebral angle subtraction.

Ranges at each inter-vertebral level were calculated as maxima and minima using the medians of these data. The overall repeatability of these ranges between 2 observers and between 2 screenings of the same subjects was calculated.

It is generally accepted that the within-subject SD at 95% range for change is the absolute measure of repeatability, sometimes called the 'coefficient of repeatability' [47,47]. With just 2 measurements per subject, this is most easily calculated as twice the square root of the mean of the squared differences between the pairs (or RMS difference). Additionally, although the analytical, or intra-observer error is usually the first source of variation considered, the physiological, or intra-subject variation is of more importance because of the need to know the repeatability of the measurement of over a short period in the same subject [49]. Furthermore, for a measurement that is meant to be suitable for clinical settings, where different observers will acquire and analyse the data, the inter-observer error supersedes and incorporates the intra-observer error. Therefore, the variances and RMS values of the differences between observers and between screenings were used to represent the inter-observer and intra-subject biological variations.

## Results

### Calibration data

The root-mean-squares of the differences between reference and computed inter-vertebral angles through 7 settings from -10° to + 20° in Calibration model 1 under optimaland degraded conditions are shown in Table 1. These results suggest that orthogonal alignment of patients is to be desired, but that inter-vertebral angle measurement at segments surrounding the one in the path of the central beam of X-rays should be sufficiently accurate to give useful information about ranges and motion patterns.

The range of error resulting from axial rotation compared with that from template marking variations is shown in Table 2. Given that rotational motion in the fixed segment model is 0°, and this error is affected more for anterior-posterior projections than for lateral ones, orthogonal radiographic positioning that minimises axial rotation is also important. This error was slightly less in the anterior-posterior projection when low kilovoltage exposure was used, but no different in the lateral projection. However, when acquired as 10-bit images as opposed to 8-bit, the mean error was 0.5° less in the anterior-posterior projection and 0.2° less in the lateral projection. Ten-bit images are therefore to be preferred.

### Exposure data

The mean exposure time for all subjects for one projection was 30 seconds (SD 2.4) including centering and acquisition. Exposure data from the 30 subjects were converted from mGy to effective dose equivalents (mSv) and are shown in Table 3 for the anterior-posterior and lateral projections. Dosages were comparable to the recommended national reference dosages for plain films [50] and are consistent with a cancer risk of between 1:10,000 and 1:100,000 [51].

### Volunteer data

**Figure 8:**
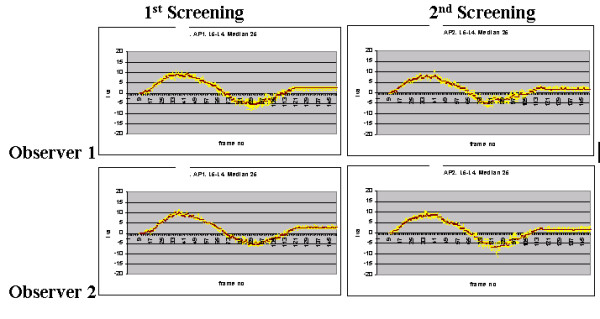
Example of L4-5 intervertebral angle series of 120 frames in sidebending; 2 screenings 30 minutes apart, analysed by 2 blinded observers. (Convention: left sidebending is +ve in graphs).

Forty-three motion segments (vertebral pairs) from L3 to L5 could be reliably tracked in side-bending motion for both first and second screenings. These provided inter-vertebral motion graphs from 43 adjacent vertebrae whose individual analyses coincided over 5 separate markings (Figure 6). Figure 8 shows an example of sidebending inter-vertebral motion graphs at the L4-5 level between observers and between screenings.

Frame-to-frame registration (tracking) failed in all of the flexion-extension sequences and additional sagittal plane screenings were subsequently obtained from 4 subjects whose images were generated at 12.5fps from a Siemens X-ray fluoroscope with digital output (DICOM). These yielded approximately 300 images per sequence. Automated registration in these image sequences yielded 13 inter-vertebral motion graphs of separate flexion and extension for comparison between 2 blinded observers. Ethical approval had not been obtained for repeated screening of these subjects, therefore intra-subject variation could not be determined.

The motion patterns were all regular and in the direction of trunk motion, but not always symmetrical, as can be seen in Figure 8. The inter-observer variation (RMS) of intervertebral rotational range was 1.86° for side-bending and 1.94° for flexion-extension. The intra-subject biological variation for side-bending range was 2.75° and 2.91° for Observers 1 and 2 respectively (Table 4).

## Discussion

The accuracy and between observer reliability found here appears to be adequate for the detection of inter-vertebral motion ranges over 3.9° (i.e. twice the worst RMS value). This raises the likelihood of OSMIA having greater diagnostic accuracy for detecting pseudathrosis than stress X-rays. Surgery to correct subtle pseudarthrosis could be better informed, or avoided altogether if OSMIA analysis can confirm solid fusion with superior sensitivity, specificity and diagnostic accuracy to plain X-rays, as initial pilot studies have suggested [52]. However, we were not always able to achieve reliable frame-to-frame registration (tracking) and in this scenario, manual registration is resorted to. In some cases, intensifier flare, poor quality images or bowel gas prevented templates from holding their vertebral outlines. Probably owing to lower contrast, automated registration of images in the lateral projection requires them not to be degraded by analogue-to-digital conversion. Nevertheless, reliability in individual subjects can be judged from the variance of the averaged inter-vertebral angles. Rarely, tracking could occur which is consistently wrong, giving misleading clinical information. To avoid this, the tracking of images can be viewed using videoclips of the frame to frame registrations to observe whether the templates are holding the image.

OSMIA can also detect paradoxical motion, irregular motion or stiffness. This might inform surgical decisions about spinal stabilisation of deformity correction, including the choice of instrumentation in individual cases. It can characterise motion patterns in terms of their regularity and symmetry, which may be useful for investigating problem back syndromes, for researching the kinematics of new flexible implants and for suspected adjacent level problems. However, any numerical analyses of these patterns would have to include the limitations imposed by the error levels found. Nevertheless, the speed at which motion segments reach the ends of their ranges may have more to do with the integrity of holding elements than the magnitudes of these ranges, according to 'neutral zone' theory [53]. Further work using symptomatic subjects with suspected loss of normal restraining capability in inter-vertebral tissues could illuminate this phenomenon.

The level of technological sophistication of the intensifier, the computer image acquisition rate, processor speed and the image bit-depth were insufficient at the time of acquiring data from asymptomatic subjects to provide valid sagittal plane motion tracking sequences. This means that we do not have intra-subject biological variation data for this plane. Such is the rate of development of fluoroscope technology that analogue outputs will eventually be replaced with digital ones, making automated frame-to-frame registration in the sagittal plane possible in all routine clinical use.

The decision about whether to collect motion data during weightbearing or in recumbency is important in the future use of OSMIA. Weightbearing motion of the spine in conscious people is more difficult to control, especially if they are in pain, and removes the possibility to exclude muscle control, allowing measurement of inter-vertebral motion patterns as determined by the disc and ligaments alone. However, it does provide patterns that include the consequences of loading, and analysis in the presence of muscle activity might sometimes be desirable. Recumbent passive motion, on the other hand, allows the trunk's motion range and regularity to be standardised, so that inter-vertebral patterns may represent only the effects of the passive holding elements. It is also more likely to be tolerable for people with pain that is aggravated by movement. This held greater promise for data collection and was therefore our starting point.

The current technique excludes translations, which are small and therefore error-prone. It also excludes axial rotations, which are not accessible with uni-planar radiography. This removes the possibility of combining the data to measure coupled and 3-dimensional motion. However, axial motions are also small, and the signal-to-noise ratio would be likely to be unacceptable even if this were possible. In the future, real-time MR could be the medium that allows this, but open coils that allow grab rates in excess of 3 frames per second are rare if available at all. Clinical examinations using MR, although radiation-free, would be much more expensive than an OSMIA assessment, where image acquisition can be done in a standard fluoroscopy room and the results analysed in a separate facility.

## Competing interests

The author(s) declare that they have no competing interests.

## Authors' contributions

The original device was conceived by AB, who served as project lead and wrote the first draft. JM wrote all codes for image analysis. FM operated the radiographic protocols and conducted most of the image analysis. All authors contributed to the study design, procedural protocols, data acquisition and analysis and the drafting of this paper.

## Pre-publication history

The pre-publication history for this paper can be accessed here:



## Figures and Tables

**Table 1:** Root-mean-squares of difference between reference and computed intervertebral angles through 7 settings from -10° to + 20° in Calibration model 1 under optimal ^1 ^and degraded ^2 ^conditions for side bending and for flexion-extension

	Side-bending		Flexion-extension	
	Optimal	Degraded	Optimal	Degraded

RMS error (°)	0.32	0.40	0.52	1.03

1. Optimal condition: X-ray beam centred on universal joint; horizontal and orthogonal to model.

2. Degraded condition: X-ray beam centred on universal joint, but angled 10° downward and whole model axially rotated by 10°.

**Table 2:** One-way analysis of variance of error in range of motion determination of a rigidly-fixed 2-vertebrae model (Calibration model 2), acquired from 5 separate screenings on the motion table through 80° in each plane, with 5 consecutive trackings of each image sequence.

	Range of error (°)	Mean squares		F-ratio	p
		Screening	Tracking		

Side-bending	1 – 4.5	4.12	0.16	25.72	<0.001
Flexion-extension	1 – 4.5	1.20	0.29	4.21	0.012

**Table 3:** Radiation dosage from 30 seconds of OSMIA lumbar spine screening compared with plain X-rays*

Projection	Effective dose (mSv) (Phantom study)		Mean Absorbed dose (Gycm^2^) Volunteer subjects (SD)	
	Males	Females	OSMIA	Plain film

Anterior-posterior	0.4	0.7	0.9 (0.45)	1.6
Lateral	0.5	0.6	1.7 (0.73)	3.0

* As recommended by the International Commission on Radiation Protection (ICRP).

**Table 4:** Inter-observer variation and intra-subject biological variation in the measurement of range of inter-vertebral motion in side-bending and flexion-extension in an asymptomatic pool of 30 male subjects aged 16–40.

	Sidebending			Flexion-extension		
	n*	RMS (°)	SD	n	RMS (°)	SD

Inter-observer variation	86^¥^	1.86	1.82	13	1.94	2.00
Intra-subject variation (Observer 1)	43	2.75	2.77	-	-	-
Intra-subject variation (Observer 2)	43	2.91	2.92	-	-	-

*n = 43 image sequences from 30 subjects for sidebending and 13 image sequences from 4 subjects for flexion-extension

^¥ ^2 observers compared range of both 1^st ^and 2^nd ^screenings
